# Treatment of bimaxillary protrusion using intra- and extra-alveolar miniscrews associated to self-ligating brackets system

**DOI:** 10.1590/2177-6709.25.5.066-084.sar

**Published:** 2020

**Authors:** Henrique Mascarenhas Villela

**Affiliations:** 1 Associação Brasileira de Odontologia - BA, Especialização e Aperfeiçoamento em Ortodontia e Ortopedia Facial (Salvador/BA, Brazil).

**Keywords:** Extra-alveolar miniscrew, Intra-alveolar miniscrew, Mini-implants, Bimaxillary protrusion, Self-ligating brackets

## Abstract

**Introduction:**

Moderate and severe bimaxillary protrusion impair the passive lip sealing and the facial and smile esthetics. The extraction of premolars can be avoided by the use of skeletal anchorage to retract both dental arches. This approach brings many advantages such as: prevents premolars extraction; simplifies orthodontic mechanics; there is no volume reduction of a premolar when smiling; control of overbite and gingival exposure. The utilization of this therapeutic approach, when associated with self-ligating brackets, can bring the possibility of spacing the appointments without damage to the treatment efficiency, and allows selection of the most appropriate torque prescriptions for each case. The intra-alveolar miniscrews are indicated for mild cases of bimaxillary protrusion, while extra-alveolar miniscrews may also be indicated for more severe cases.

**Objective:**

To report the treatment of three cases of mild, moderate and severe bimaxillary protrusion, in which intra- and extra-alveolar miniscrews were used, according to the retraction required.

**Conclusion:**

The retraction of both upper and lower dental arches using orthodontic intra- and extra-alveolar miniscrews, associated with self-ligating brackets, is an excellent tool to correct mild to severe bimaxillary protrusion, thus reducing the need of premolar extraction and simplifying the orthodontic management.

## RESUMO

**Introdução:**

As biprotrusões moderadas e severas dificultam o selamento labial passivo, comprometem a estética facial e do sorriso. As extrações de pré-molares podem ser evitadas quando se utiliza a ancoragem esquelética como recurso para retrair ambas as arcadas. Essa abordagem traz as seguintes vantagens: evita que o paciente seja submetido ao processo da extração dos pré-molares; simplifica a mecânica ortodôntica; não reduz o volume de um pré-molar de cada lado no sorriso; e auxilia no controle da sobremordida e da exposição gengival. A utilização dessa terapêutica, quando associada aos aparelhos autoligáveis, possibilita maiores intervalos entre as consultas, sem comprometer a eficiência do tratamento, e permite a seleção dos torques mais adequados para essa mecânica. Os miniparafusos intra-alveolares podem ser utilizados na correção de biprotrusões mais suaves, enquanto os extra-alveolares podem ser indicados, também, nos casos mais severos.

**Objetivo:**

Relatar os tratamentos de três casos clínicos de biprotrusão leve, moderada e severa, respectivamente, efetuando a retração das arcadas em uma única fase, utilizando miniparafusos intra- e extra-alveolares, conforme a magnitude da retração necessária.

**Conclusão:**

A retração das arcadas superior e inferior com miniparafusos ortodônticos intra- e extra-alveolares associados aos aparelhos autoligáveis é um excelente recurso para a correção das biprotrusões de suave a severa, diminuindo a necessidade de extrações de pré-molares e simplificando a mecânica ortodôntica.

## INTRODUCTION

Patients with dentoalveolar bimaxillary protrusion usually present increase in incisor inclination, accompanied by lip protrusion, which may cause muscle imbalance with lip incompetence.^1^ ^,^ ^2^ One of the premises to achieve a good facial esthetics is good positioning and shape of the lips.^3^ The retraction of incisors, in this malocclusion, promotes reduction of their inclinations and improvement in soft tissues, altering the profile.^4^ ^,^ ^5^ The amount of retraction of anterior teeth and movement of the lips are important factors to predict the change in facial profile after orthodontic treatment.^2^ ^,^ ^3^

Often, extractions of first premolars are indicated to provide space for anterior retraction and improve the inclination of incisors in their bone bases.^6^ ^,^ ^7^

Temporary anchorage devices (TADs), such as miniplates, can offer an excellent anchorage option for full retraction of the arch to correct crowding or protrusion.^8^

With the advent of orthodontic miniscrews, the possibility to promote tooth movements supported on fixed points in the oral cavity, minimizing undesired side effects, made the treatments more efficient and predictable, reducing the need for patient compliance and simplifying the orthodontic mechanics.^9^ ^-^ ^15^ Orthodontic miniscrews can provide special benefits for the treatment of mild or moderate bimaxillary protrusion, such as the possibility of retracting the entire arch to reduce the incisor inclination, decreasing the indication of premolar extractions.^9^ ^,^ ^10^

Correction of mild bimaxillary protrusion can be achieved by full retraction of the arches in a single stage, using intra-alveolar miniscrews, which are placed in the region between first molars and second premolars. This positioning between the roots limits the amount of retraction, due to the little space available between roots in this area.^16^ Other sites for placement of intra-alveolar miniscrews have been used to achieve more spaces for greater retractions, such as in the region between first and second molars or distal to the lower second molars.^9^ ^,^ ^17^ Two-stage retraction, with intra-alveolar miniscrews, can also be used for retraction in corrections of more severe bimaxillary protrusion. This strategy consists of changing the screws, placing them more distally, when the second premolar root is close to the screw body.^18^

In 2007, Liou et al.^19^ proposed a method for screw placement on the infrazygomatic crest (IZC), in the buccal region of first molars. These authors suggested a more inclined placement of the miniscrew, to allow greater sagittal correction without interference from the screw body with the mesiobuccal root of the upper first molar, which allows total retraction of the maxillary arch in a single stage.

In 2008, Villela et al.^20^ used a titanium miniscrew in the region between upper first and second molars, with greater inclination in their placement, aiming at removing the screw body from the molar roots, which allowed greater retraction of the upper arch in a single stage.

Chang et al.^21^ ^-^ ^23^ used extra-alveolar stainless steel screws, with larger diameter and length, in areas of denser cortex, both in the infrazygomatic crest region and in the mandibular buccal shelf, by the use of a distalization and retraction mechanics of the entire arch in a single stage. This strategy can be used to compensate Class II and Class III malocclusions and bimaxillary protrusion, reducing the indication of extractions.

Self-ligating brackets do not require the use of metallic or elastic ligatures to retain the orthodontic arch.^24^ Self-ligating appliances have reduced friction compared to conventional brackets, since they do not require the use of ligatures.^25^ ^-^ ^28^ Also, they promote a decrease in the accumulation of dental plaque, less injury to oral tissues, shorter chair time, and allow longer intervals between consultations.^29^ ^,^ ^30^ As an aid in torque control, brackets with different prescriptions can have different torque values, which can be used individually for each type of orthodontic movement desired. The available prescriptions are high, low and standard torque.^24^ ^,^ ^31^

Thus, the objective of the present study is to demonstrate the efficiency of intra- and extra-alveolar miniscrews associated with self-ligating brackets in the treatment of mild, moderate and severe bimaxillary protrusion.

## BIOMECHANICS USED FOR TOTAL RETRACTION OF ARCHES WITH ORTHODONTIC MINISCREWS

**Figure 1:**
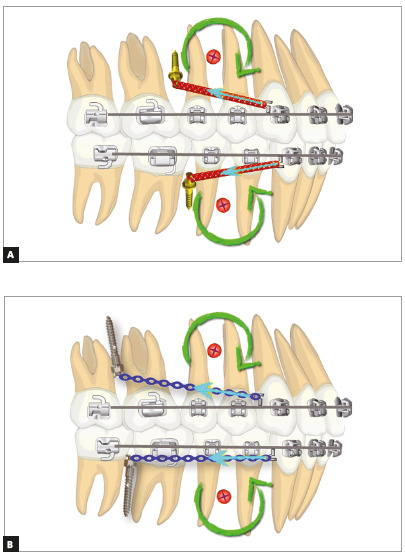
Biomechanics of total retraction of the arches using intra-alveolar (A) and extra-alveolar (B) miniscrews.

When performing full retraction of the arches, there is a tendency to rotation around their center of resistance, which is positioned between the premolars, at the level of the middle third of roots, both in the maxilla and mandible. When the retraction is anchored on miniscrews, the line of force action passes more occlusally to the center of resistance. This line is determined by two points, which are the sites where the power elements are attached (hook and screw head). The effects of this retraction produce retroclination of incisors, with a tendency to extrusion, and distalization of posterior teeth, with a tendency to intrusion. In cases of bimaxillary protrusion in which extrusion of the upper incisors is not desired, it is important to use short anterior hooks. The head of miniscrews must be positioned closer to the mucogingival line, to produce an inclined line of force action and perform retraction with an intrusion component in the anterior teeth (Fig 1).

## CASE REPORTS

Three mesofacial patients will be presented, with balanced facial thirds, reduced overjet and overbite, lack of passive lip sealing and mild, moderate and severe bimaxillary protrusion, respectively, treated with self-ligating brackets associated with four orthodontic miniscrews. The mild bimaxillary protrusion was treated with intra-alveolar miniscrews; moderate and severe cases were treated with extra-alveolar screws.

### CASE REPORT 1

#### Description and diagnosis

**Figure 2:**
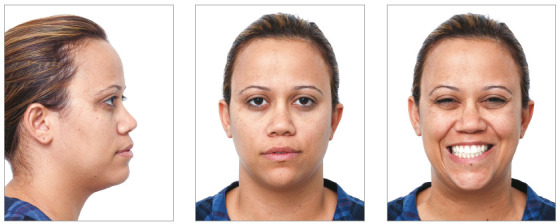
Initial extraoral views.

Female patient, aged 30 years, reported dissatisfaction with the protrusion of teeth and lips. The frontal facial analysis showed symmetry, balanced facial thirds, good proportion between facial height and width, characteristics of mesofacial individuals. The lateral facial analysis revealed a Pattern I face, with good convexity, well-positioned maxilla and mandible. Lateral evaluation of the lower facial third evidenced increased projection of the lips, which compromised the facial esthetics. Evaluation of smile revealed that the upper arch presented good exposure of the upper incisors and gingiva, with excess exposure of lower incisors and asymmetry of the lower lip. It also revealed a good vertical relationship between the upper incisors and upper lip (Fig 2).

**Figure 3:**
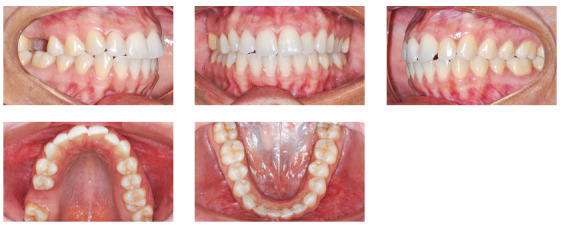
Initial intraoral views of the patient with Class I malocclusion, bimaxillary protrusion and reduced overjet and overbite.

Analysis of dental arches showed Angle Class I malocclusion, with excellent molar, premolar and canine sagittal relationships; however, with absence of the upper right first molar. Non-coincident upper and lower dental midlines were observed, due to deviation of the upper midline to the left because of a greater crowding of tooth #22 and contra-angulation of tooth #21. The overjet and overbite were reduced, due to the increased inclination of upper and lower incisors (Fig 3). The upper arch presented moderate crowding and rotation of teeth #11, #21 and #22. The lower arch presented good alignment and leveling, with presence of a fixed canine-to-canine retainer on the lingual aspect, due to a previous orthodontic treatment (Fig 3).

**Figure 4:**
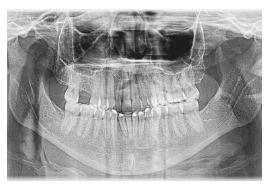
Initial panoramic radiograph.

Analysis of the panoramic radiograph showed absence of the upper right first molar and upper and lower third molars. The other teeth and periodontal structures were in normal condition (Fig 4).

**Figure 5:**
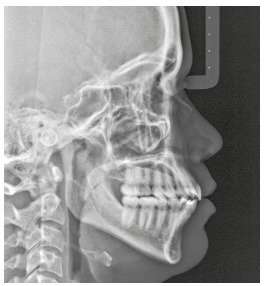
Initial lateral cephalogram.

The initial cephalometric analysis revealed good positioning of the maxilla and mandible, slightly divergent angles of the palatal, occlusal and mandibular planes, and normal lower facial height, characteristic of mesofacial individuals (Fig 5).

**Figure 6:**
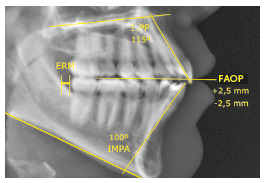
Initial cephalometric measurements.

» The upper incisors had a slightly increased inclination (1.PP=115°). This angle assesses the relationship between the long axis of incisors and the palatal plane, with a mean normal value of 110°.
» The upper incisors had a good vertical relationship with the upper lip, with a FAOP (Functional Aesthetic Occlusal Plane) of 2.5 mm. The lower incisors required extrusion of 2.5 mm to touch the FAOP plane (FAOP=+2.5 mm/-2.5 mm). The FAOP evaluates the positioning relationship between molars, incisors and upper lip stoma^32^. The normal measure is 2.0 to 4.0 mm with the upper incisor. The lower incisor must be tangent to this plane.
» The lower incisors had a slightly increased inclination (IMPA=100°). This angle assesses the relationship between long axis of lower incisors and the mandibular plane, and the normal measure is 90°.
» Retromolar space is the space between the distal aspect of the crown of the lower second molar and the mesial aspect of the mandibular ramus. This space must be compatible with the need for distalization (Fig 6). There was good space in the retromolar region (RMR) to perform distalization of the lower arch.

#### Treatment planning and mechanics employed

The orthodontic treatment planning consisted of alignment and leveling the arches and subsequent retraction, with anchorage on intra-alveolar orthodontic miniscrews, which were positioned between the first molars and second premolars, with the objective of reducing the protrusion and inclination of incisors and consequently improving facial and smile esthetics.

Interactive self-ligating brackets, 0.022-in slot, with MBT prescription were used. This prescription was selected due to the greater torque in upper incisors (central incisors + 17° and lateral incisors + 10°) to obtain greater torque control during retraction, since the upper incisors needed small reduction in their inclination. In the lower arch, torques are reduced (lower incisors -6°), with less torque control, to allow greater reduction of inclination during retraction.

**Figure 7:**
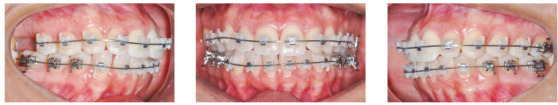
Intermediate thermoactivated NiTi rectangular archwires.

The alignment of the upper and lower arches was initiated with 0.014-in thermoactivated NiTi archwires, followed by 0.018-in; 0.014 x 0.025-in and 0.018 x 0.025-in (Fig 7).

**Figure 8:**
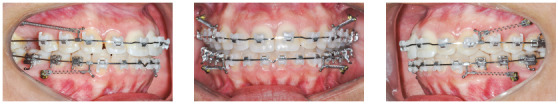
Initial stage of retraction of the upper and lower arches, with 0.019 x 0.025-in stainless steel archwire, with intra-alveolar miniscrews placed between the second premolars and first molars.

The retraction of both arches began with the 0.019 x 0.025-in stainless steel archwires, anchored in orthodontic miniscrews positioned between the second premolars and first molars, on the buccal side. The ideal initial force for total retraction of the arch is 250g/cm² and it should gradually be increased in the following consultations, up to a maximum of 400g^33^ ^,^ ^34^. This calibration was performed by reducing the spring length. On the upper right side, a milder force was used due to absence of the first molar, offering less resistance to distalization in this hemiarch. The miniscrews used (SIN, Sistema de Implante Nacional S.A., São Paulo/SP) had 1.6-mm diameter, 8.0-mm body length and 1.0-mm transmucosal profile. They were placed at 8.0 mm towards the apex in relation to the main archwire in the upper arch, and at 7.0 mm in the lower arch. This positioning was performed according to the band of keratinized mucosa, which is narrower in the mandible than in the maxilla. The miniscrews were inserted in the mucogingival line (which separates the keratinized from the alveolar mucosa). They were placed with an inclination of 80 to 90° in relation to the cortical plate in the maxilla and more inclined in the mandible (Fig 8).

After six months of retraction, a mild reduction in the inclination of incisor crowns was clinically observed, with consequent reduction in inclination of the lips. This allowed greater interaction by the patient during treatment, who could assess the gradual alteration of the profile and give an opinion on the best time to complete the arches retraction - unlike with premolar extractions, in which total space closure is necessary and often requires changing the anchorage strategy for mesialization of posterior teeth, when the retraction of anterior teeth is no longer desired.

**Figure 9:**
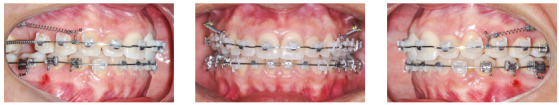
Intermediate stage of retraction of the upper arch, with 0.019 x 0.025-in stainless steel archwire, and completion of lower retraction.

Retraction of the lower arch was performed faster than that of the upper arch, generating an increased overjet. At that moment, the screws in the mandible were removed and the retraction was continued in the upper arch (Fig 9).

**Figure 10:**
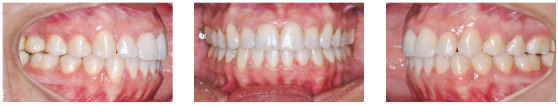
Intraoral view of the patient with canines, premolars and molars on the left side in key of occlusion. On the right side, an implant-supported denture was placed with dimensions compatible with an upper third premolar. The incisor relationship was normalized.

After three months of upper retraction, the overjet was normalized, and the canine relationships finalized with an key of occlusion. At that moment, the implant was placed in the region of the upper first molar. After the osseointegration period, the crown was placed on the implant (Fig 10).

#### Results

**Figure 11:**
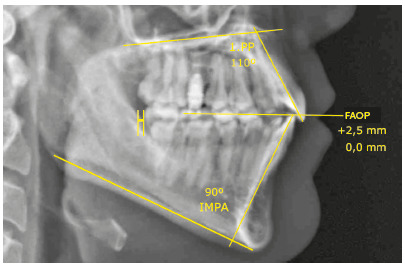
Final cephalometric measurements.

Simultaneous retraction of the arches anchored on orthodontic miniscrews was able to retract the upper incisors in 2.3 mm and reduce their inclination by 5° (1.PP=110°). The lower incisors retracted 3.0 mm and reduced their inclination by 10° (IMPA=90°). The upper incisors, despite the retraction and reduction of inclination, maintained their relationship with the FAOP at 2.5 mm. This fact was due to retraction with intrusion vector. The lower incisors were also retracted, with a reduction in inclination; however, they extruded and touched the FAOP (FAOP = +2.5 mm/0.0 mm). Canines, premolars and molars ended in an key of occlusion. The incisor relationship improved, increasing the overjet and overbite (Fig 11).

**Figure 12:**
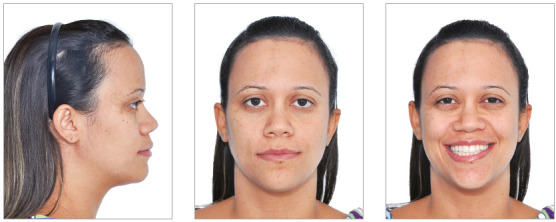
Final extraoral views.

In the facial aspect, there were small positive changes, with a slight reduction in lip projection, compatible with the small reduction in incisor inclination (Fig 12).

**Figure 13:**
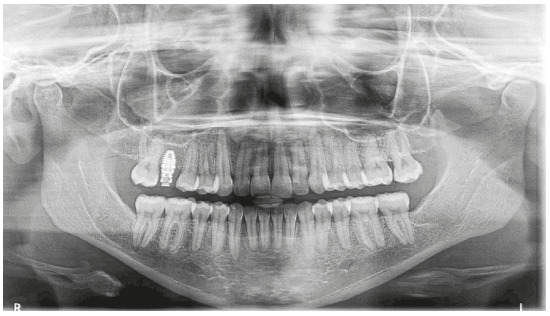
Final panoramic radiograph.

Analysis of the final panoramic radiograph did not show any significant alteration in relation to the initial radiograph, except for implant placement in the region of the upper right first molar (Fig 13).

**Figure 14:**
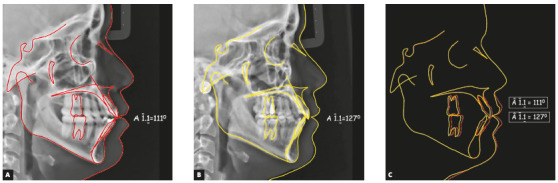
Initial (A), final (B) and superimposition (C) lateral cephalograms.

Cephalometrically, the most relevant changes were reduction of bimaxillary protrusion and inclination of the upper and lower incisors; distalization of all posterior teeth; maintenance of vertical dimension; and improvement of soft tissue esthetics. There was a 16° reduction in the interincisal angle, changing from 111° to 127° (Fig 14).

### CASE REPORT 2

#### Description and diagnosis

Female patient, aged 27 years, reported dissatisfaction with protruding teeth and lack of passive lip sealing. The frontal facial analysis revealed symmetry, a good proportion between facial height and width and balanced facial thirds, characteristic of mesofacial individuals. The lateral analysis revealed a Pattern I face with good convexity and well-positioned maxilla and mandible. Lateral evaluation of the lower facial third did not reveal increased projection of the upper lip, but interposition of the upper incisors between the lips. This projection of incisors impaired passive lip sealing and promoted lower lip eversion, which compromised the facial esthetics.

**Figure 15:**
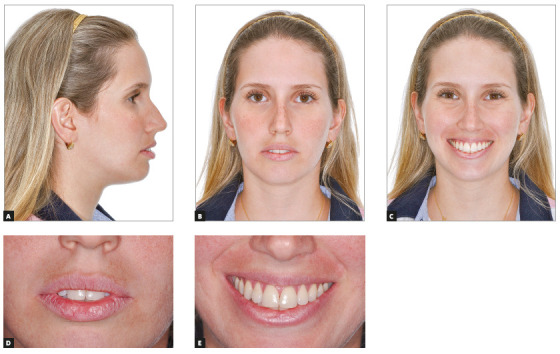
A-C) Initial extraoral views. D, E) Close views of the lips, at rest and smiling.

In the evaluation of smile, the upper arch presented good vertical exposure of upper incisors and some gingiva. It also revealed increased exposure of incisors with the lips at rest (Figs 15A, 15B and 15C). In a closer view, it was possible to observe the interference of upper incisors on the lips, with increased inclination, both at rest and when smiling, compromising the esthetics (Figs 15D and 15E).

**Figure 16:**
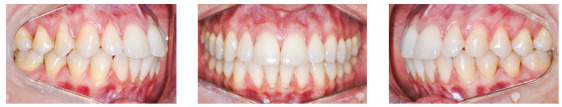
Initial intraoral views of the patient with Class I malocclusion, bimaxillary protrusion and reduced overjet and overbite.

Analysis of the dental arches revealed an Angle Class I malocclusion, with excellent sagittal relationships of molars, premolars and canines, coincident upper and lower dental midlines, mild crowding in the lower arch and decreased overjet and overbite, due to the increased inclination of upper and lower incisors (Fig 16).

**Figure 17:**
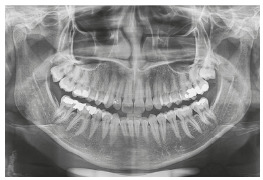
Initial panoramic radiograph.

The panoramic radiographic analysis showed the absence of lower third molars and the presence of upper third molars. The upper left third molar was mesially angulated and impacted on the second molar. Then, extractions of the upper third molars were requested. The other teeth and periodontal structures had normal conditions (Fig 17).

**Figure 18:**
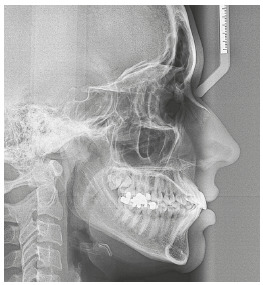
Initial lateral cephalogram.

The initial cephalometric analysis revealed a slight maxillary deficiency and good mandibular positioning; slightly divergent angles of the palatal, occlusal and mandibular planes; and normal height of the lower facial third, characteristic of mesofacial individuals (Fig 18).

**Figure 19:**
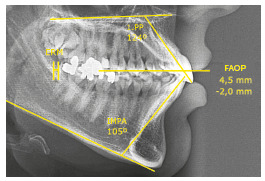
Initial cephalometric measurements.

» Upper incisors had increased inclination (1.PP=124°).
» Upper incisors had a slightly increased vertical relationship with the upper lip, namely 4.5 mm; however, the lower incisors required extrusion of 2.0 mm to touch the FAOP plane (FAOP=4.5 mm/-2.0 mm).
» Lower incisors had increased inclination (IMPA=105°).
» Presence of reduced space in the retromolar region (RMR), yet sufficient to distalize the lower arch (Fig 19).

#### Treatment planning and mechanics employed

The orthodontic treatment planning consisted of alignment and leveling, with subsequent retraction of the arches, anchored on extra-alveolar orthodontic miniscrews, which were positioned between the first and second molars, to reduce the protrusion and inclination of incisors, with consequent improvement of facial function and esthetics.

Passive self-ligating brackets (Easy Clip Plus, Aditek do Brasil Ltda), with 0.022-in slot, standard Damon prescription were used. This prescription was chosen for the upper and lower incisors and canines. In the upper arch, the values ​​were: central incisors +15°, lateral incisors +6° and canines +7°. The upper teeth required a small reduction in their inclination, with little control during retraction. It should be remembered that the working archwire used for retraction is 0.019 x 0.025-in stainless steel, and that there is a gap of nearly 12° between the arch and bracket slot.^32^ If greater torque control was necessary, without the need to reduce the inclination, the prescription of choice would be high torque (central incisors +22°, lateral incisors +13° and canines +11°). In the lower arch, the torques in the incisors are more reduced (incisors -3° and canines +7°); the prescription of +7° in the canines prevents exaggerated lingual inclination of these teeth during retraction, helping in the transverse control.

**Figure 20:**
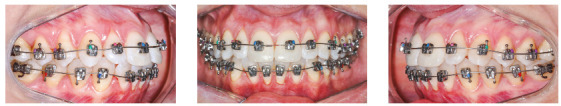
Initial 0.014-in thermoactivated NiTi archwires.

The alignment of upper and lower arches was started, with 0.014-in thermoactivated NiTi archwires; followed by 0.018-in and 0.014 x 0.025-in (Fig 20).

**Figure 21:**
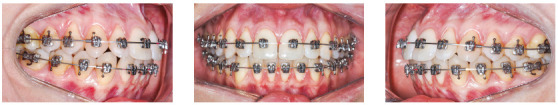
0.018 x 0.025-in rectangular intermediate thermoactivated NiTi archwires.

In the 0.018 x 0.025-in archwire, after complete alignment and leveling of the arches, interproximal stripping was performed on the upper and lower incisors, to improve the anatomy of crowns, which had triangular shape and dark spaces in the papilla spaces, known as black spaces (Fig 21).

**Figure 22:**
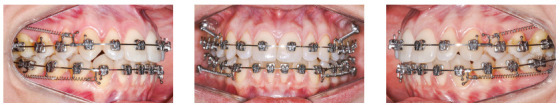
Initial stage of retraction of the upper and lower arches, with 0.019 x 0.025-in stainless steel archwire, with extra-alveolar miniscrews placed between the first and second molars.

The retraction of both arches started with the 0.019 x 0.025-in stainless steel archwires anchored on extra-alveolar orthodontic miniscrews. In the maxilla, the screws were positioned in the region of the infrazygomatic crest (IZC), in the mesial aspect of upper second molars, on the buccal side. In the mandible, the screws were placed between the second molars and first molars on the buccal side, in the region known as buccal shelf. These two sites have a greater amount of cortical bone and the screws are inserted as vertically as possible. This strategy aims at positioning the body as far from the roots as possible to allow greater sagittal corrections. The initial force used was 250 g/cm², increased in the following consultations, by reducing spring length. Stainless steel screws (Bioray, New Taipei City, Taiwan), with 2.0-mm diameter, 2.0-mm transmucosal profile and 10.0-mm body length were placed (Fig 22).

**Figure 23:**
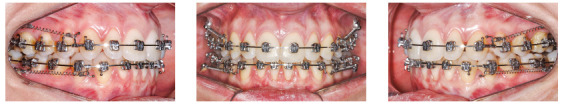
Final stage of retraction of upper and lower arches, with 0.019 x 0.025-in stainless steel archwire.

After eight months of retraction, a reduction in incisor inclination was clinically observed, with consequent improvement in overjet and overbite. The lips then showed passive lip sealing (Fig 23).

#### Results

**Figure 24:**
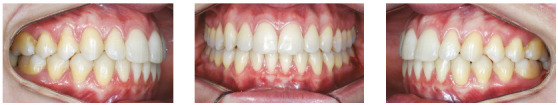
Intraoral views of the patient with canines, premolars and molars in key of occlusion and normal relationship of incisors.

At treatment completion, the upper incisors were retracted with vertical control, without extrusion. The lower incisors reduced the inclination and extruded. Canines, premolars and molars ended in key of occlusion (Fig 24).

**Figure 25:**
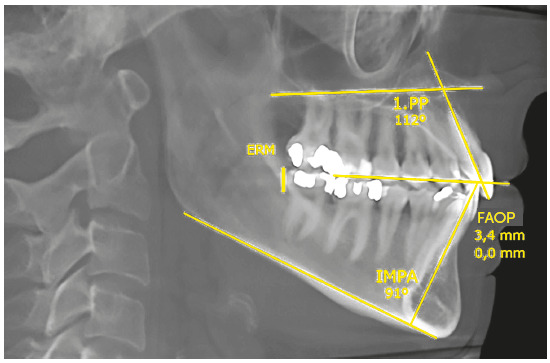
Final cephalometric measurements.

Simultaneous traction of the arches with anchorage on extra-alveolar screws allowed retraction of upper incisors in 5.0 mm and reduced their inclination by 12° (1.PP=112°). The lower incisors retracted 5.5 mm and reduced their inclination by 14° (IMPA=91°). The relationship between incisors improved, increasing the overjet and overbite. The upper incisors, despite the retraction and reduction of inclination, improved their relationship with the FAOP, going to 3.5 mm. This was due to retraction with an intrusion vector. The lower incisors were also retracted with reduced inclination; however, they extruded and touched the FAOP (FAOP=3.5 mm/0.0 mm) (Fig 25).

**Figure 26:**
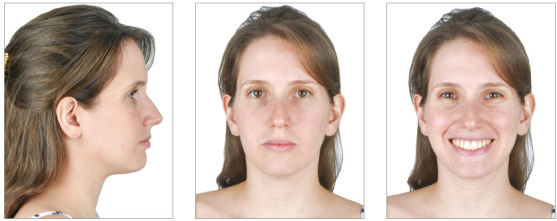
Final extraoral views.

In the facial aspect, there were positive changes, with reduced incisor inclination, which allowed passive lip sealing and a more harmonious smile. However, retraction of the upper incisors reduced the exposure of the upper lip vermillion, which is not a positive aspect (Fig 26).

**Figure 27:**
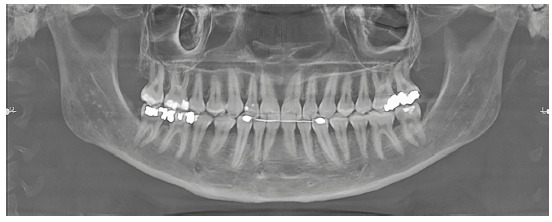
Final panoramic radiograph.

Analysis of the final panoramic radiograph showed good parallelism of the roots and without the upper third molars. The other periodontal structures maintained normal conditions (Fig 27).

**Figure 28:**
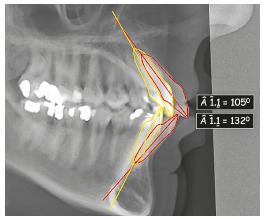
Superimposition of the tooth movement performed and comparison of the change in the interincisal angle.

Cephalometrically, the most relevant changes were reduction of bimaxillary protrusion and inclination of the upper and lower incisors, distalization of all posterior teeth, maintenance of vertical dimension and improvement of soft tissue esthetics. There was a 27° reduction in the interincisal angle, changing from 105° to 132° (Fig 28).

### CASE REPORT 3

#### Description and diagnosis

A 36-year-old female patient reported great dissatisfaction with the protrusion of teeth and lack of passive lip sealing. The frontal facial analysis showed symmetry, balanced facial thirds and a good proportion between facial height and width, characteristic of mesofacial individuals. The lateral analysis revealed a Pattern I face, with good convexity, well-positioned maxilla and mandible. Lateral evaluation of the lower facial third revealed exaggerated projection of the upper and lower lips, with interposition of upper incisors between them. This increased incisor inclination prevented passive lip sealing and compromised the facial esthetics.

**Figure 29:**
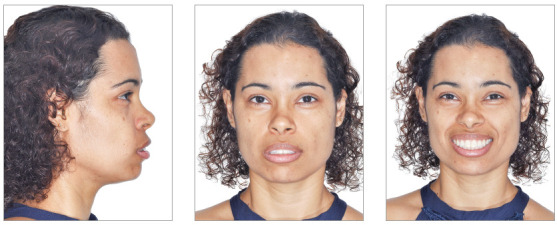
Initial extraoral views.

When evaluating the smile, the upper arch presented good vertical exposure of the upper incisors and some gingiva. Despite this good vertical relationship, the smile was unpleasant, due to the exaggerated inclination of incisors. There was also increased exposure of incisors with the lips at rest (Fig 29).

**Figure 30:**
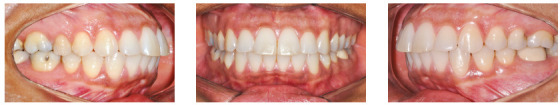
Initial intraoral views of the patient with Class I malocclusion, bimaxillary protrusion and reduced overjet and overbite.

The analysis of dental arches revealed Angle Class I malocclusion, with excellent sagittal relationships of molars, premolars and canines, coincident upper and lower dental midlines, aligned dental arches, without crowding, and decreased overbite and overjet, due to the increased inclination of upper and lower incisors (Fig 30).

**Figure 31:**
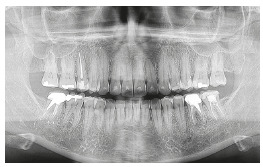
Initial panoramic radiograph.

The analysis of panoramic radiograph showed absence of upper and lower third molars. The other teeth and periodontal structures had normal conditions (Fig 31).

**Figure 32:**
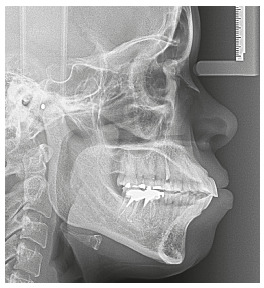
Initial lateral cephalogram.

The initial cephalometric analysis revealed good positioning of the maxilla and mandible; slightly divergent angles of the palatal, occlusal and mandibular planes; and normal height of the lower facial third, characteristic of mesofacial individuals (Fig 32).

**Figure 33:**
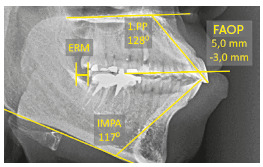
Initial cephalometric measurements.

» Upper incisors presented increased inclination (1.PP=128°).
» Upper incisors had a slightly increased vertical relationship with the upper lip, of 5.0 mm; however, the lower incisors needed to extrude 3.0 mm to touch the FAOP plane (FAOP=+5.0 mm/-3.0 mm).
» Lower incisors presented increased inclination (IMPA=117°).
» Presence of good space in the retromolar region (RMR), sufficient to distalize the lower arch (Fig 33).

#### Treatment planning and mechanics employed

The orthodontic treatment planning consisted of alignment and leveling with subsequent retraction of the arches, with anchorage on extra-alveolar orthodontic miniscrews. These screws were positioned between the first and second molars to reduce the protrusion and inclination of incisors and consequently improve the function and facial esthetics.

Passive self-ligating brackets Easy Clip Plus (Aditek do Brasil Ltda., Cravinhos/SP, Brazil) were used, with 0.022-in slots, standard Damon prescription. This prescription was chosen for the upper and lower incisors and canines. The upper teeth required great reduction in inclination. It was planned to achieve this reduction by retraction, rather than by reduced torque.

**Figure 34:**
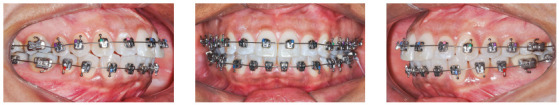
Initial 0.014-in initial thermoactivated NiTi archwires.

The alignment of upper and lower arches was initiated with 0.014-in thermoactivated NiTi archwires, followed by 0.014 x 0.025-in and 0.018 x 0.025-in archwires (Fig 34).

**Figure 35:**
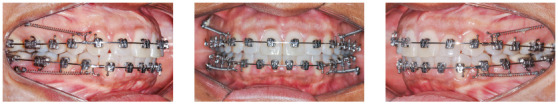
Initial stage of retraction of upper and lower arches, with 0.019 x 0.025-in stainless steel archwire, with extra-alveolar miniscrews placed between the first and second molars.

Retraction of the two dental arches began with 0.019 x 0.025-in stainless steel archwires anchored on extra-alveolar orthodontic miniscrews, which were positioned on the infrazygomatic crest (IZC) and on the buccal shelf. The initial force used for total retraction of the arches was 250g/cm², gradually increased in the following consultations, by reducing the spring length. After 8 months of retraction, the springs were replaced by medium elastomeric chains, to generate more retraction force, used for another 6 months, adding up to 14 months of retraction. The extra-alveolar miniscrews placed were made of stainless steel, (DAT Steel, São Bernardo do Campo/SP, Brazil), with 2.0-mm diameter, 2.0-mm transmucosal profile and 10.0-mm body length (Fig 35).

**Figure 36:**
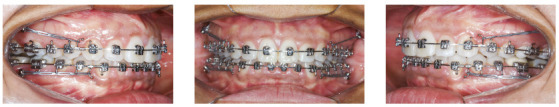
Final stage of retraction of the upper and lower arches, with 0.019 x 0.025-in stainless steel archwire.

After 14 months of retraction, a reduction in incisor inclination was clinically observed, with consequent improvement in overjet and overbite. The lips started to show passive sealing (Fig 36).

#### Results

**Figure 37:**
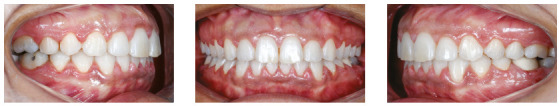
Intraoral views of the patient with canines, premolars and molars in key of occlusion, and normal relationship of incisors.

At treatment completion, the upper incisors retracted, with slight intrusion, due to vertical control. The lower incisors reduced the inclination and extruded, normalizing the overjet and overbite. Canines, premolars and molars ended in key of occlusion (Fig 37).

**Figure 38:**
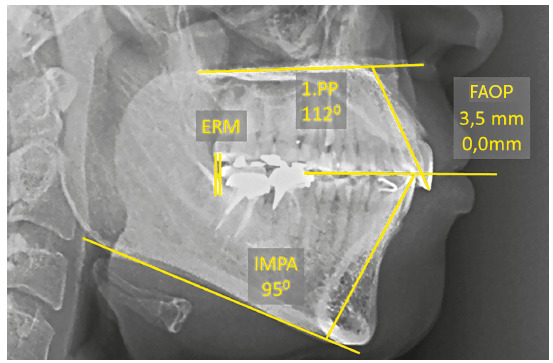
Final cephalometric measurements.

Simultaneous traction of the arches with anchorage on the extra-alveolar screws managed to retract the upper incisors in 7.0 mm and reduced their inclination by 16° (1.PP=112°). The lower incisors retracted 8.5 mm and reduced their inclination by 26° (IMPA=91°). The upper incisors, despite the retraction and reduction of inclination, improved their relationship with the FAOP, changing to 3.5 mm. This was due to retraction with an intrusion vector. The lower incisors were also retracted, with reduction in their inclination; however, they extruded and touched the FAOP (FAOP +3.5 mm/0.0 mm) (Fig 38).

**Figure 39:**
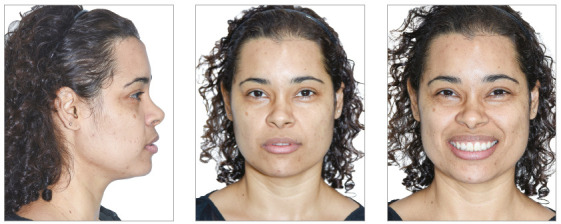
Final extraoral views.

 In the facial aspect, there were positive changes, with reduced incisor inclination, which allowed passive lip sealing and a more harmonious smile. Despite the great retraction, the lips continued with increased volume, due to their greater thickness (Fig 39).

**Figure 40:**
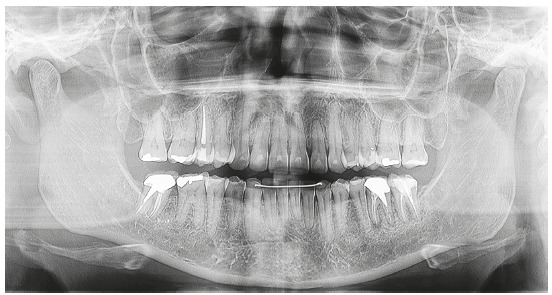
Final panoramic radiograph.

Analysis of the final panoramic radiograph showed good parallelism of the roots. The other periodontal structures maintained normal conditions (Fig 40).

**Figure 41:**
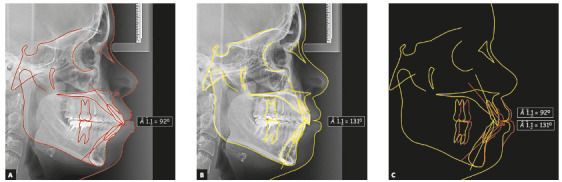
Initial (A), final (B) and superimposition (C) of lateral cephalograms, illustrating the change that occurred in the interincisal angle.

Cephalometrically, the most relevant changes were reduction of bimaxillary protrusion and inclination of the upper and lower incisors, distalization of all posterior teeth, maintenance of vertical dimension, and improvement of soft tissue esthetics. There was a reduction of 39° in the interincisal angle, from 92° to 131° (Fig 41).

## DISCUSSION

The facial esthetic results resulting from anterior retractions varied according to the amount of retraction of incisors and lip thickness. According to the study of Hayashida et al.^3^, the results are influenced by the ethnic background.

Total retraction of the arches without extractions using skeletal anchorage was able to achieve great movements and may be more efficient than conventional mechanics, with extraction of first premolars. This strategy provided retractions of up to 8.5 mm of the lower incisors, as shown in Case 3. Willians and Hosila^35^ concluded that, in cases involving extraction of the first four premolars, approximately 66.5% of the available extraction space was occupied by retraction of the anterior segment. In the present clinical cases, we decided to perform retraction of both arches, with the aid of orthodontic screws associated with self-ligating appliances, instead of extracting four premolars. This is an excellent treatment option, since it does not reduce the volume of first premolars in the arches, maintaining the transverse volume of smile. According to Ong and Woods,^36^ the general average reduction of the arch perimeter was 11.3 mm with extractions of premolars. However, previous studies^37^ ^,^ ^38^ have shown that, in cases of association of crowding with dental protrusions, these extractions should be indicated.

The association of intra- and extra-alveolar screws with self-ligating brackets and thermoactivated archwires reduces the number of consultations, since the archwires can be changed every two months. This fact does not compromise the efficiency nor increases the overall treatment time.^14^ ^,^ ^15^ ^,^ ^18^ ^,^ ^39^

The variable prescriptions in these cases of bimaxillary protrusion, in which the median or standard torque was used, do not present great advantages over other prescriptions, since a great control of incisor torque was not required because the side effect of incisor retroclination during retraction of the arches was desired. Only the torques of upper and lower canines should be 7° positive, to inhibit the tendency of lingual inclination of these teeth during retraction.

The intra-alveolar miniscrews can be used as anchorage in the treatment of cases with mild bimaxillary protrusion, due to the limited space between the roots. However, it is possible to increase the amount of retraction by changing the screw position, to continue distalization and achieve greater movements.^26^

The extra-alveolar miniscrews proved to be an excellent anchorage option in the treatment of moderate to severe bimaxillary protrusion, avoiding the extraction of premolars. The magnitude of retraction occurred according to the time of use of the mechanics, since the body of screws was not an obstacle for root movement. Total retraction of the arches in the correction of severe bimaxillary protrusion lasted 14 months, while in moderate bimaxillary protrusion it lasted 8 months. The retraction of upper incisors required an intrusion component to maintain a good vertical relationship with the lips. The lower incisors were extruded to improve the overbite. The understanding and skill of biomechanics in the use of skeletal anchorage is necessary to achieve more predictable and desirable results.

The disadvantage of this type of approach is the need to use skeletal anchorage devices, which requires specific knowledge of the professional, both for placement and control of biomechanics. In addition, the use of miniscrews can raise resistance in patients, since it is an invasive procedure.

This approach reduces the indication of extraction of first premolars; however, it requires space distal to the second molars to achieve the total retraction of the arches. This leads to a frequent request for extraction of third molars, which is better accepted by patients, since it does not compromise the esthetics and their removal is usually indicated.

Thermoactivated NiTi archwires have important characteristics in the initial treatment stage, as they respond differently when subjected to low or high tension. These archwires, when submitted to small deflections, present an excellent elastic recovery; however, when they are subjected to large deflections, resulting from irregular positioning of the teeth, they become more flexible, dissipating a milder force. As the teeth move and the irregularities decrease, the tension decreases and the elastic recovery capacity increases, becoming a little less flexible. This property allows maintenance of this type of archwire for a longer time in the initial treatment stages, since they release more constant forces during the process of correcting tooth irregularities. This reduces the need for monthly archwire changes and the number of different archwire sizes. When associated with self-ligating brackets, which do not require the monthly replacement of elastic ligatures, they allow patients to stay longer with the mechanics installed and reduce the number of consultations.

## CONCLUSION

The self-ligating brackets system associated with skeletal anchorage with intra- and extra-alveolar miniscrews proved to be efficient in correcting mild, moderate and severe bimaxillary protrusion, with improved lip posture, without reducing the volume of the first premolars in the arches and consequently maintaining the transverse volume of the smile. This strategy can bring some advantages, such as: decreased indication of premolar extractions; reduced need of patient compliance; simplification of orthodontic mechanics; and simplified placement and removal of screws. Self-ligating appliances, together with high-tech thermoactivated archwires used in the initial treatment stages, can reduce the number of consultations, which can be more spaced, without compromising the results or increasing the overall treatment time.
